# Qingkailing Suppresses the Activation of BV2 Microglial Cells by Inhibiting Hypoxia/Reoxygenation-Induced Inflammatory Responses

**DOI:** 10.1155/2014/696218

**Published:** 2014-04-28

**Authors:** Lulu Mana, Shan Wang, Haiyan Zhu, Yanwei Xing, Lixia Lou, Aiming Wu, Bin Dong, Yikun Sun, Shuo Yang, Lin Wang, Yonghong Gao

**Affiliations:** ^1^Key Laboratory of Chinese Internal Medicine of Ministry of Education and Beijing, Dongzhimen Hospital Affiliated to Beijing University of Chinese Medicine, Beijing 100700, China; ^2^The Southern District of Guang'anmen Hospital, China Academy of Chinese Medical Sciences, Beijing 102618, China; ^3^Guang'anmen Hospital, China Academy of Chinese Medical Sciences, Beijing 100053, China; ^4^Institute of Information on Traditional Chinese Medicine, China Academy of Chinese Medical Sciences, Beijing 100700, China; ^5^Department of Preventive Health Care, China-Japan Friendship Hospital, Beijing 10029, China

## Abstract

Qingkailing (QKL) is a well-known composite extract used in traditional Chinese medicine. This extract has been extensively administered to treat the acute phase of cerebrovascular disease. Our previous experiments confirmed that QKL exerts an inhibitory effect on cerebral ischemia-induced inflammatory responses. However, whether QKL suppresses the activation of microglia, the primary resident immune cells in the brain, has yet to be determined. In this study, BV2 microglial cells were used to validate the protective effects of QKL treatment following ischemia-reperfusion injury simulated via hypoxia/reoxygenation *in vitro*. Under these conditions, high expression levels of ROS, COX-2, iNOS, and p-p38 protein were detected. Following ischemia/reperfusion injury, QKL significantly increased the activity of BV2 cells to approximately the basal level by modulating microglial activation via inhibition of inflammatory factors, including TNF-**α**, COX-2, iNOS, and p-p38. However, QKL treatment also displayed dose-dependent differences in its inhibitory effects on p38 phosphorylation and inflammatory factor expression.

## 1. Introduction

Ischemic cerebrovascular disease (ICVD) is a notably common clinical cerebrovascular disease. The mechanisms underlying cerebral ischemia-reperfusion injury are quite complicated. Oxidative stress and the inflammatory response are considered to be the key mediators of cerebral ischemic injury. The injury and repair mechanisms in anoxic and ischemic environments, which are mediated by the abnormally activated microglia, have become a recent target of neurological research field. Unfortunately, these mechanisms have yet to be fully elucidated. The specific mechanism by which microglia become activated in response to cerebral ischemia remains controversial [1, 2]. When ischemia occurs, microglia are rapidly activated initially in response to the stressful stimulus. Studies have revealed that activated microglia can protect neurons via phagocytic removal of pathogens and dead cell debris, as well as secretion of neurotrophic factors [3–5].

However, additional evidence has indicated that activated microglia can also synthesize and release inflammatory mediators, leading to neuronal dysfunction and death, in response to injury and stimulation [6, 7]. Abnormally activated microglia can damage neurons by releasing proinflammatory cytokines, inflammation-related enzymes, and chemokines and activating NADPH oxidase to generate reactive oxygen species, induce the expression of IL-1*β*, TNF-*α* and MMP-9, promote leukocyte infiltration, and weaken the blood-brain barrier [8]. Because of their rapid activation and their important roles in oxidative stress and the inflammatory response, microglia may represent a new line of investigation in the search for a clinical therapy for cerebral ischemia.

Qingkailing (QKL) is a modified extract from a traditional Chinese medicine, An-Gong-Niu-Huang Pill. It is composed of Radix Isatidis, Flos Lonicerae, Concha Margaritifera Usta, baicalin, Fructus gardeniae, cholic acid, hyodeoxycholic acid, and Cornu Bubali [9]. QKL injection has been widely used clinically to treat the acute phase of cerebrovascular disease and has displayed excellent efficacy in improving neurological function [10, 11]. We and other investigators demonstrated that QKL injection can improve neurological function, decrease the expression of adhesion molecules, inhibit inflammatory responses, and partially restore BBB function in a rat model of brain ischemia/reperfusion injury [12–15].

Based on the findings described above, we speculated that QKL might modulate the inflammatory response mediated by activated microglia. BV2 microglial cells were used as a model, and* in vitro* hypoxia/reoxygenation was performed to simulate ischemia/reperfusion* in vivo*. The roles of the inflammatory factors TNF-*α*, iNOS, and COX-2 and the regulatory function of the corresponding signaling pathways are also examined.

## 2. Materials and Methods

### 2.1. Materials

MTT and dimethyl-sulfoxide (DMSO) were obtained from Solarbio Corporation, China. The mouse TNF-*α* ELISA Kit was purchased from Neobioscience Co., Ltd., China. Trizol reagent was purchased from Invitrogen Corporation, USA. The Reverse Transcription System (A3500 kit) was purchased from Promega Corporation, USA. The Power SYBR Green PCR Master Mix kit was purchased from ABI Corporation, USA. RIPA Lysis Buffer, Super ECL Plus Detection Reagent, Prestained Protein Marker (10-170KD), proteinase inhibitor cocktail, PhosSTOP phosphatase inhibitor, and the ROS detection kit (product no: C1300) were purchased from Applygen Technologies Co., Ltd. COX-2 (H-62) and iNOS (N-20) were purchased from Santa Corporation, USA. The mouse anti-*β*-actin and HRP-labeled goat anti-rabbit IgG antibodies were purchased from Wuhan Boster Biological Technology, Ltd. The p-p38 and p38*α*/*β* (H-147) antibodies were purchased from Beijing ZhongShan-Golden Bridge Biological Technology Co., Ltd., China. Minocycline was purchased from Sigma Corporation, USA. QKL was purchased from Yabao Beizhongda (Beijing) Pharmaceutical Co., Ltd., China.

### 2.2. Cell Culture and the Hypoxia-Reoxygenation Injury Model

#### 2.2.1. Cell Culture

Mouse microglial BV2 cells were purchased from the cell culture center at the Institute of Basic Medical Sciences (IBMS) of the Chinese Academy of Medical Sciences (CAMS). The cells were suspended in culture medium specialized for microglia (DMEM containing 10% fetal bovine serum, 100 U/mL penicillin, and 100 U/mL streptomycin). At 80% confluency, the cells were passaged using trypsin-EDTA solution (0.05% trypsin and 0.02% EDTA).

#### 2.2.2. Establishment of the* In Vitro* BV2 Cell Hypoxia/Reoxygenation Model

BV2 cells were digested into a cell suspension, and the cell density was adjusted to 5 × 10^4^ cells/mL. After 80% confluence, the cells were washed 3 times with PBS, replaced with serum-free DMEM medium, and placed in a three-gas incubator at 37°C containing 1.0% O_2_ to initiate hypoxia, followed by reoxygenation in an incubator at 37°C containing 5% CO_2_.

#### 2.2.3. Evaluation of the BV2 Cell Viability

The MTT method was used to assess cell viability. Cell viability was measured using an enzyme-labeling instrument at 492 nm. The hypoxia duration was 12 hours, while reoxygenation occurred for 12 or 24 hours.

#### 2.2.4. ROS Assay Using Flow Cytometry

The BV2 cells were cultured in a six-well plate at a density of 5 × 10^4^ cells/mL. The cells were digested and harvested following exposure to normoxia or 12 h of hypoxia followed by 12 h of reoxygenation. Then, the cells were incubated in 1 mL of DCFH-DA (10 *μ*mol/L) for 30 minutes. The cells were washed three times with PBS and analyzed via flow cytometry (Beckman Co., FC50).

### 2.3. Effects of QKL on BV2 Cell Viability after Hypoxia/Reoxygenation

#### 2.3.1. Cell Group

The experiment comprised six groups: a control group (the cells were cultured normally in serum-free DMEM); the model group (12 h of hypoxia and 12 h of reoxygenation in serum-free DMEM medium); the low, medium, and high QKL groups (a final QKL concentration of 0.0625%, 0.125%, or 0.25%, resp., in serum-free DMEM was added before hypoxia); and the minocycline group (200 nmol/L minocycline was added before hypoxia).

#### 2.3.2. MTT Assay the Changes of Cell Viability

The hypoxia duration was 12 hours, while reoxygenation lasted for 12 hours. The MTT method was used to assess cell viability, as described above.

### 2.4. Effect of QKL on the Concentration of TNF-*α* in the Supernatant of BV2 Cells Subjected to Hypoxia/Reoxygenation

The groups assigned were the same as above. The cell supernatants were collected and stored at −80°C. ELISA was performed to measure the TNF-*α* concentration in the cell culture supernatants.

### 2.5. Effect of QKL on Inflammatory Cytokine (COX-2 and iNOS) mRNA Expression in BV2 Cells Subjected to Hypoxia/Reoxygenation

Real-time quantitative polymerase chain reaction (real-time PCR) was performed to evaluate the mRNA expression of COX-2 and iNOS. The mRNA was extracted, reverse-transcribed, and quantified via real-time PCR. The following PCR primers were used: COX-2 (191 bp), forward: 5′-GATGACTGCCCAACTCCC-3′; reverse: 5′-AACCCAGGTCCTCGCTTA-3′. iNOS (95 bp), forward: ′-CAGCTGGGCTGTACAAACCTT-3′; reverse: 5′-CATTGGAAGTGAAGCGTTTCG-3′. *β*-actin (185 bp), forward: 5′-AGGCCAACCGTGAAAAGATG-3′; reverse: ′-TGGCGTGAGGGAG A GCATAG-3′.

The cycling conditions were 50°C for 2 min and initial denaturation at 95°C for 10 min, followed by 40 cycles at 95°C for 30 s and 55°C for 30 s. The data were analyzed using the Stratagene MX3000P System. The levels of COX-2 and iNOS mRNA normalized to that of *β*-actin were compared between the different groups.

### 2.6. Effect of QKL on Inflammatory Factor (COX-2 and iNOS) Protein Expression in BV2 Cells and on the p38 Signaling Pathway during Ischemia-Reperfusion Injury* In Vitro*

After reoxygenation, the cells were washed 3 times with PBS, followed by pipetting repeatedly in RIPA Lysis Buffer. Then, the cellular protein extract was collected. The proteins were separated via 10% SDS-PAGE and transferred to nitrocellulose membranes, which were subsequently incubated in a primary antibody to COX-2, iNOS, p-p38, or p38 (1 : 1000) at 4°C overnight. After the membranes were washed three times with TBST, they were further incubated in the appropriate horseradish peroxidase-conjugated secondary antibody for 2 hours at room temperature. Then, ECL visualization was performed. NIH Image J was used to calculate the gray values. The intensity of the target protein was normalized to that of an internal reference to determine the relative expression level of the target protein.

### 2.7. Statistical Analysis

The data were expressed as the means ± SD. Statistical analysis was performed via one-way analysis of variance (ANOVA) using SPSS11.0 software. *P* < 0.05 was considered to be significant.

## 3. Results

### 3.1. Establishment of the* In Vitro* Hypoxia/Reoxygenation Model

**Figure 1:**
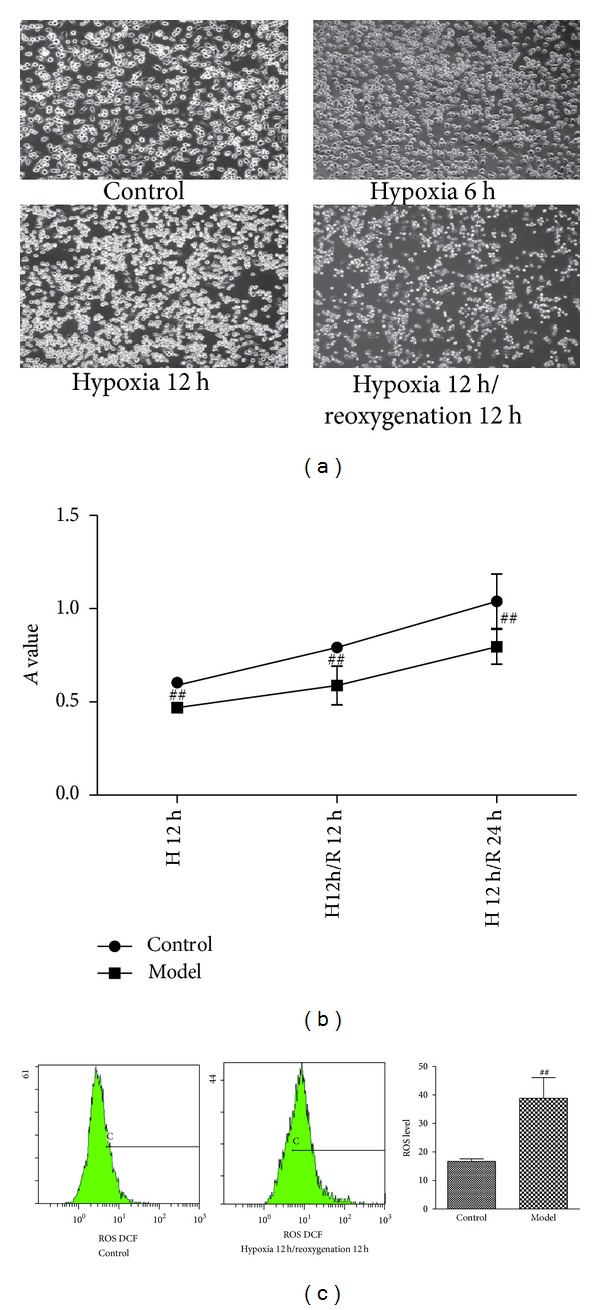
Effects of different durations of hypoxia/reoxygenation on BV2 microglia cells. (a) BV2 cells were exposed to hypoxia for 6 or 12 hours followed by reoxygenation for 12 hours. Morphological changes were observed under an inverted microscope (×100). (b) After exposure to hypoxia for 12 hours, the BV2 cells were reoxygenated for 12 or 24 hours. Cell viability was assed using the MTT method. Each value indicates the mean ± SD. *N* = 5.  ^##^ *P* < 0.01 compared to the control group. (c) ROS level in BV2 microglial cells after hypoxia for 12 hours and reoxygenation for 12 hours based on flow cytometry. *N* = 4.  ^##^ *P* < 0.01 compared to the control group.

Based on inverted phase contrast microscopy, approximately 60–80% of the cells in the control group were well-anchored contained, a polygonal cell body; approximately 20–40% of the cells were suspended. As hypoxia continued, the cell body gradually became rounded, and the attached cells became suspended and gathered into clusters. Hypoxia for 12 h resulted in significant changes compared to the control group, and these changes were exacerbated after reoxygenation for 24 h (Figure 1(a)). MTT colorimetry suggested that, compared to the control group, after hypoxia for 12 h followed by reoxygenation for 12 h or 24 h in an three-gas incubator, the number of surviving BV2 cells in the model group were significantly reduced (*P* < 0.01) (Figure 1(b)). As shown in Figure 1(c), the level of ROS in the model group (hypoxia for 12 h and reoxygenation for 12 h) was significantly higher than the normal group (*P* < 0.01).

Based on the comparisons of the cellular morphology and viability after different hypoxia durations, we chose 12 h of hypoxia and 12 h of reoxygenation, which caused significant injury, for the simulation of ischemia-reperfusion injury* in vitro.*

### 3.2. QKL Increased Cell Viability in BV2 Microglia Exposed to 12 Hours of Hypoxia and 12 Hours of Reoxygenation

**Figure 2:**
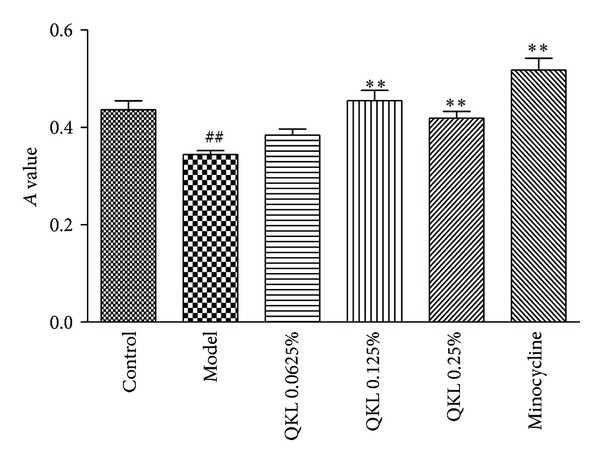
Effects of different dosages of QKL on the viability of BV2 microglial cells in the hypoxia/reoxygenation model. The cells were treated with 0.0625%, 0.125%, or 0.25% QKL or 200 *μ*M minocycline before hypoxia. The MTT method was performed, and the results are expressed as the means ± SD; *n* = 6.  ^##^ *P* < 0.01 compared to the control group; **P < 0.01 compared to the model group.

Based on the model establishment above, according to the results of pilot experiments, the nontoxic dose range of QKL treatment was chosen to be 0.0625%, 0.125%, and 0.25% (Figure 2). Alternatively, 200 nmol/L minocycline, the specific inhibitor of microglia, was applied prior to hypoxia. The results revealed that the *A* value of the cells treated with any of the three concentrations of QKL or minocycline, which were exposed to hypoxia for 12 h and reoxygenation for 12 h, was significantly higher than the model group. This result confirmed that QKL remarkably increases BV2 cell survival and activation under ischemia-reperfusion conditions, reverting them to control levels, preventing both the activation and injury of BV2 cells.

### 3.3. QKL Decreased the Expression of TNF-*α* in BV2 Cell Supernatants

**Figure 3:**
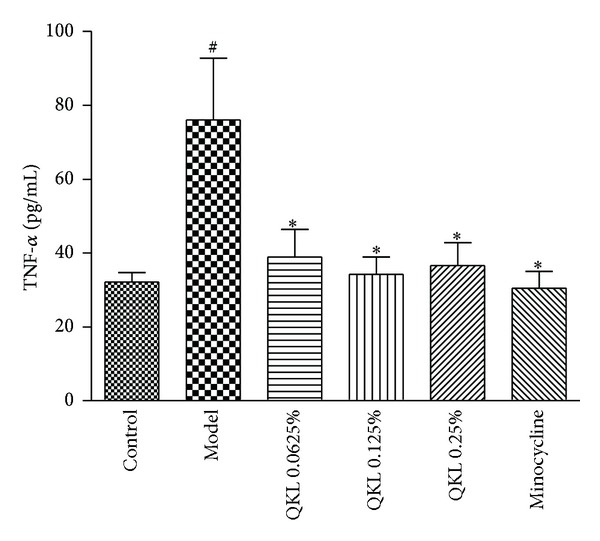
Effect of QKL on the expression of TNF-*α* in BV2 cell supernatants. Cell supernatants were collected after hypoxia/reoxygenation. ELISA was performed to measure the expression of TNF-*α*; the results are expressed as the means ± SD. *N* = 6.  ^##^ *P* < 0.01 compared to the control group; **P < 0.01 compared to the model group.

TNF-*α* is an important inflammatory factor that is released after microglial activation that plays an important role in nerve inflammation. The results (Figure 3) revealed that compared to the control group, the concentration of TNF-*α* in the model group was significantly increased (*P* < 0.05). Treatment with 0.0625%, 0.125%, or 0.25% QKL or 200 nmol/L minocycline significantly reduced TNF-*α* production induced by hypoxia/reoxygenation compared to the model group (*P* < 0.05). Together, these results indicated that ischemia-reperfusion injury activates BV2 cells to produce a large quantity of TNF-*α*, which participates in the inflammatory response induced by ischemia reperfusion. QKL blocked microglial activation by inhibiting TNF-*α* expression.

### 3.4. QKL Decreased the Expression of COX-2 and iNOS

**Figure 4:**
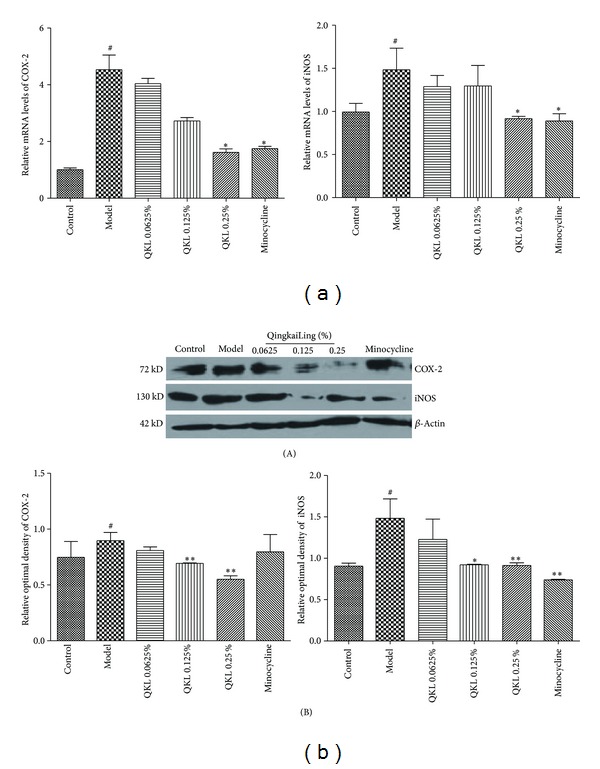
Hypoxia/reoxygenation-induced changes in the protein and mRNA expression levels of COX-2 and iNOS; *β*-actin was also quantified as a control. (a) The mRNA expression levels of COX-2 and iNOS were determined via real-time PCR. (b) The cellular protein expression levels of COX-2 and iNOS were analyzed via Western blot. ^#^ *P* < 0.01 compared to the control group; *P < 0.05 and **P < 0.01 compared to the model group.

Activated microglia can also damage neurons by releasing proinflammatory cytokines, inflammation related enzymes, and so on. We performed real-time PCR and Western blot analyses to examine the changes in the expression of COX-2 and iNOS (Figures 4(a) and 4(b)). After hypoxia for 12 hours and reoxygenation for 12 hours, the mRNA and protein expression levels of COX-2 and iNOS were significantly increased. Treatment with 0.25% QKL or 200 nmol/L minocycline significantly decreased the mRNA expression level compared to the model group. However, treatment with 0.25% QKL clearly decreased the protein expression of COX-2 and iNOS in BV2 cells exposed to hypoxia/reoxygenation. In addition, treatment with 0.125% QKL also decreased the protein expression of iNOS in BV2 cells. Together, these results demonstrate that QKL alleviates the increases in COX-2 and iNOS expression, protecting against further ischemia-reperfusion caused by protein and gene expression. Importantly, these results are consistent with those of the cell viability and TNF-*α* expression assays described above.

### 3.5. QKL Inhibited p38 Phosphorylation in BV2 Cells Exposed to Hypoxia/Reoxygenation

**Figure 5:**
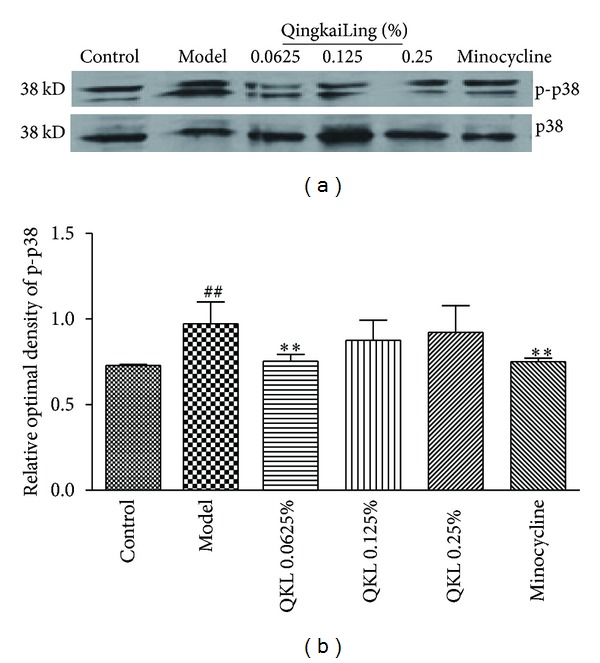
Effect of QKL on p38 phosphorylation in BV2 microglial cells exposed to hypoxia/reoxygenation. (a) Western blot was performed on the cell lysates using anti-p-p38 and anti-p38 antibodies. (b) The differences in the protein expression levels between the groups were analyzed using Image J software. ^##^ *P* < 0.01 compared to the control group. **P < 0.01 compared to the model group.

To further elucidate whether the p38 signaling pathway affects the expression of iNOS and COX2 in BV2 cells, we measured the expression of phosphorylated p38 protein and analyzed the ratio of p-p38 to p38. As shown in Figure 5, the control group expressed a small amount of p-p38 protein, and p-38 expression was significantly increased in the model group. Treatment with 0.0625% QKL or 200 nmol/L minocycline significantly decreased p-p38 protein expression in BV2 cells subjected to hypoxia/reoxygenation compared to the model group (*P* < 0.01).

## 4. Discussion

As one of the primary mechanisms of cerebral ischemia, the inflammatory response can cause secondary brain injury. Inflammatory cytokines, which are released from so-called inflammatory cells such as neutrophils, granulocytes, lymphocytes, and glia, can stimulate the adherence of leukocytes to the vascular endothelium, induce inflammatory responses, and cause tissue edema, destroying brain tissue [3–5].

Previous studies have demonstrated that, whether under the conditions of diseases or culture systems, the primary source of proinflammatory cytokines is microglia rather than astrocytes. Moreover, IL-1*β* and TNF-*α* have been suggested to be the most important proinflammatory cytokines in this process [16]. The massive expression of TNF-*α* acts as a key mediator of cerebral ischemia-induced inflammation, which cannot only mediate a cascade of inflammatory responses but also amplify inflammation by interacting with other inflammatory factors, thus inducing the expression of adhesion molecules on the endothelium as well as other inflammatory mediators by macrophages, endothelial cells, and spongiocytes. TNF-*α* induces the migration of activated leukocytes to the ischemic area, and these cells release free radicals, causing secondary injury [17, 18]. Furthermore, TNF-*α* itself can enhance the activation of microglia [19, 20].

Based on the findings of our study, after hypoxia for 12 h followed by reoxygenation for 12 h, the levels of ROS increased and the concentration of TNF-*α* in the cell culture supernatant was significantly increased, but cell viability decreased. Treatment with the high, middle, or low concentration of QKL clearly inhibited the expression of TNF-*α* and increased cell viability.

iNOS and COX-2 are suggested to be important factors involved in microglial activation and critical mediators of cerebral ischemic injury caused by microglia. When reperfusion occurs, a large amount of oxygen molecules gathers in the cerebral ischemia area, which react with NO to produce powerful oxidants that can cause irreversible oxidative damage to cells [21]. An iNOS gene knock-out mouse model exhibited a significant decrease in the infarct volume following middle cerebral artery occlusion [22]. NADPH is a multisubunit enzyme complex; once NADPH becomes activated, ROS, a second messenger of cell signal transduction, is strongly produced, which leads to the activation of the p38-MAPK signaling pathway and the expression of proinflammatory cytokines (e.g., TNF-*α*) and inflammation-related enzymes (e.g., iNOS and COX-2) [23, 24]. Conversely, NADPH is specifically activated by these cytokines, thus promoting the production of ROS and other inflammatory factors. In this manner, a vicious cycle of ever-increasing neurotoxins is initiated, ultimately resulting in neuronal necrosis [25].

In our experiments, increases in the mRNA and protein expression levels of both COX-2 and iNOS in BV2 microglia were induced by hypoxia/reoxygenation. Treatment with the low or middle concentration of QKL displayed a significant effect on the protein expression of COX-2. The inhibitory effects of the high concentration of QKL on the mRNA and protein expression of iNOS and COX-2 were the most significant, which demonstrated that the inhibitory effects of QKL on the inflammatory response became stronger as the QKL concentration increased.

Studies have indicated that oxidative stress, TNF-*α*, and so forth [4], via the p38 MAPK signaling pathway in conjunction with other signaling pathways, modulate the gene expression and activity of enzymes in response to cell stress, and it is hypothesized that protein phosphorylation is necessary for the activation of these pathways and the progression of signal transduction [26]. In our study, the low concentration of QKL significantly reduced the expression of p-p38 protein in BV2 cells subjected to hypoxia/reoxygenation. However, this effect is not equivalent to its effect on the expression of levels of COX-2 and iNOS. Together, these results indicate that QKL exerts different actions at varying concentrations. The high concentration of QKL significantly inhibited microglial activation, but it may also affect other signaling pathways. However, there were dose-dependent differences in the inhibition of p38 phosphorylation and inflammatory cytokine expression that merit further examination.
